# Global nursing in an Ebola viral haemorrhagic fever outbreak: before, during and after deployment

**DOI:** 10.1080/16549716.2017.1371427

**Published:** 2017-10-10

**Authors:** Eva von Strauss, Stéphanie Paillard-Borg, Jessica Holmgren, Panu Saaristo

**Affiliations:** ^a^ Department of Public Health and Medicine, The Swedish Red Cross University College (SRCUC), Stockholm, Sweden; ^b^ Department of Nursing and Care, The Swedish Red Cross University College (SRCUC), Stockholm, Sweden; ^c^ Health and Care Department, International Federation of Red Cross and Red Crescent Societies (IFRC), Geneva, Switzerland

**Keywords:** Ebola Virus Disease, disaster, global health, global health care, global nursing, nursing education, preparedness, training, viral haemorrhagic fever

## Abstract

**Background**: Nurses are on the forefront and play a key role in global disaster responses. Nevertheless, they are often not prepared for the challenges they are facing and research is scarce regarding the nursing skills required for first responders during a disaster situation.

**Objectives**: To investigate how returnee nursing staff experienced deployment before, during and after having worked for the Red Cross at an Ebola Treatment Center in Kenema, West Africa, and to supply knowledge on how to better prepare and support staff for viral haemorrhagic fever outbreaks.

**Methods**: A descriptive, cross-sectional approach. Questionnaires were administered to nurses having worked with patients suffering from Ebola in 2014 and 2015. Data collection covered aspects of pre-, during and post-deployment on clinical training, personal health, stress management, leadership styles, socio-cultural exposure and knowledge transfer, as well as attitudes from others. Data was analysed using both quantitative and qualitative methods.

**Results**: Response-rate was 88%: forty-four nurses from 15 different countries outside West Africa answered the questionnaire. The respondents identified the following needs for improvement: increased mental health and psychosocial support and hands-on coping strategies with focus on pre- and post-deployment; more pre-deployment task-oriented clinical training; and workload reduction, as exhaustion is a risk for safety.

**Conclusions**: This study supplies knowledge on how to better prepare health care staff for future viral haemorrhagic fever outbreaks and other disasters. Participants were satisfied with their pre-deployment physical health preparation, whereas they stressed the importance of mental health support combined with psychosocial support after deployment. Furthermore, additional pre-clinical training was requested.

## Background

The Ebola Virus Disease (EVD), formerly known as Ebola haemorrhagic fever, is a severe illness with a fatality rate of up to 90%. The virus was first identified in 1976 when two simultaneous outbreaks occurred: one in a village not far from the Ebola River in the Democratic Republic of Congo (DRC), and the other in a remote area of Sudan [1]. The origin of the virus is unknown, but current evidence suggests that fruit bats (Pteropodidae) may be a host to the virus [2]. In February 2014, for the first time in West Africa, a case of EVD was confirmed in the Republic of Guinea. The outbreak then quickly spread to neighbouring countries: Sierra Leone, Liberia, Nigeria, Senegal, DRC and Mali [3]. This outbreak is the largest and most complex EVD outbreak ever; by March 2016, there had been 28,616 cases of EVD and 11,310 deaths reported worldwide [4].

In response to the outbreak, the Centers for Disease Control and Prevention (CDC) activated its Emergency Operations Center to coordinate activities with other US government agencies, the World Health Organization (WHO) and other domestic and international partners [5]. An emergency is generally considered to be a serious, unexpected and often dangerous situation requiring immediate action, and in August 2014, the WHO declared the West Africa outbreak a Public Health Emergency of International Concern [6,7]. The International Federation of Red Cross and Red Crescent Societies (IFRC) is the largest humanitarian network in the world, and its staff and volunteers play an active role in responding to humanitarian crises worldwide. This was the first large-scale clinical response to a viral haemorrhagic fever (VHF) outbreak that the IFRC responded to by recruiting up to 80 international nurses to set up their first Ebola Treatment Center (ETC) in Kenema, Sierra Leone. Although the IFRC’s Emergency Response Units (ERU) [8] had previously been active in epidemic responses (i.e. cholera treatment centres in acute watery diarrhoea outbreaks), the ERU system did not have, at the time, a pre-defined configuration for this type of clinical work, nor was VHF case management part of the training. During the EVD outbreak in West Africa, the IFRC worked closely with the local and national Red Cross Societies, Ministries of Health, WHO and Médecins Sans Frontières (MSF). Red Cross volunteers also provided psychosocial support to families affected by the outbreak, and assisted in the management of the burials of dead bodies [9].

**Figure 1.**
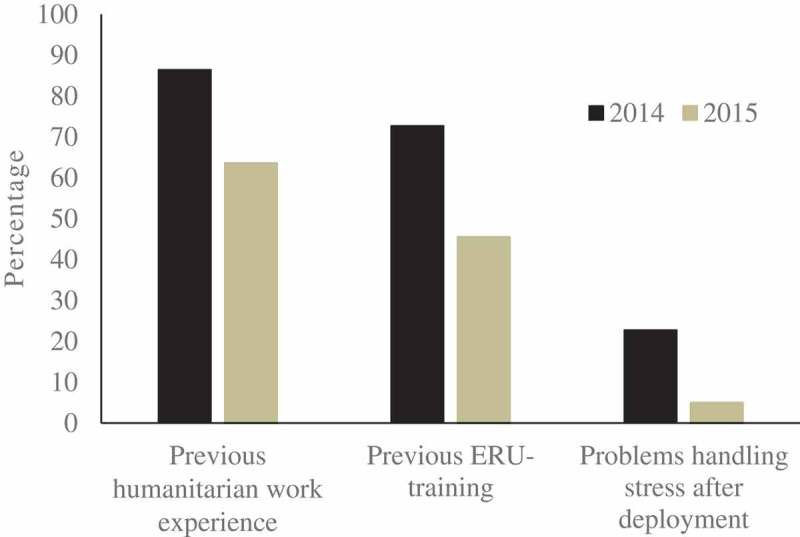
Percentage of study population having previous humanitarian work experience, previous emergency response unit (ERU) training, and having problems handling stress after deployment divided by time of deployment (during 2014 or 2015 respectively).

**Figure 2.**
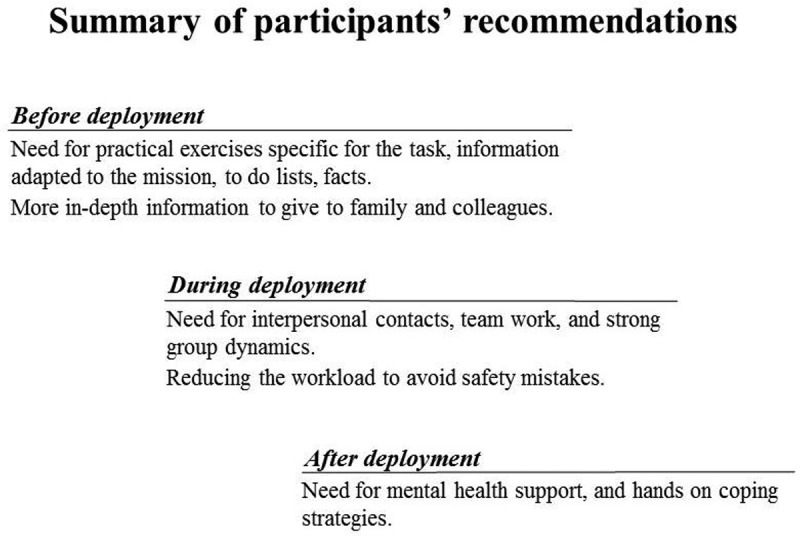
A summary of the participants’ recommendations on how to better prepare health care staff for future viral haemorrhagic fever (VHF) outbreaks before, during and after deployment.

The IFRC defines disaster as a sudden, calamitous event that seriously disrupts the functioning of a community or society and causes human, material and economic or environmental losses that exceed the community’s or society’s ability to cope using its own resources. Though often caused by nature, disasters can have human origins [10]. Nurses are on the forefront of global disaster responses and often play a key role. Nevertheless, they are seldom prepared for the challenges they are facing, and research is scarce regarding the required nursing skills for first responders needed during a disaster situation [11,12]. Previous research concerning EVD has mainly focused on guidance for health care workers, such as protective equipment and behaviours [13], infection control and emergency management [14–18], set up of community care centers [19], as well as research in laboratory settings [20,21]. Few studies have focused on health care staff’s own experiences when caring for patients under very extreme conditions [22–25]. The fact that EVD is a highly lethal and contagious disease contributed to the fear and stigma attached to it [9,26,27]. Health-care workers have been infected while caring for patients with Ebola [28,29]. This could happen through close contact with patients when there were rigorous infection precautions lacking, as well as through burial ceremonies where mourners had direct contact with the body of the deceased person [3,27].

Increased globalisation has led to enhanced awareness of nurses as being major contributors to global health care [30,31] i.e. health issues that have the potential to directly or indirectly transcend national, governmental and geographical borders [32]. Nurses employed by humanitarian non-governmental organisations are recurrently exposed to risks of infections and even life-threatening dangers. In addition, EVD, considered as an emerging infectious disease [33], challenges the medical world, as well as nurses working with global nursing, because of their relatively limited experience of it.

The main objective of the present study was to investigate how returnee nursing staff experienced their deployment before, during and after having worked for the Red Cross at an ETC in Kenema, West Africa, during a VHF outbreak. The second objective was to supply knowledge on how to better prepare health care staff for future VHF outbreaks and other disasters.

## Methods

### Study design

A descriptive cross-sectional approach with a possibility for longitudinal follow-up.

### Study population

In a case of humanitarian disaster, the IFRC triggers a rapid alert system using Global System for Mobile communications (GSM) and emails sent to its National Societies having ERUs available. Team members are then deployed to the affected area within 48 hours. The majority of the team members have had previous field experience and are all professionally qualified, and are often active volunteers within the Red Cross while having a regular job. During the Ebola outbreak in West Africa, the IFRC set up two ETCs in Sierra Leone: the first in Kenema and then a second in Kono.

Inclusion criteria for this study were nurses having worked with patients suffering from EVD at the ETC in Kenema during the outbreak between 1 March 2014 and 30 September 2015. An invitation letter was sent to 78 nurses; among them 13 were returned as ‘unknown’ email addresses. Of the remaining 65, we were able to contact 50 persons, and six of them (12%) refrained from participation. Thus, 44 nurses (88%) responded to the questionnaire.

One participant was in Kenema during March 2014, 21 participants between August and December 2014, 20 participants between January and May 2015, and two between August and September 2015. The average time between the last day of their deployment and their response to the questionnaire was 139 days, ranging from 5 to 542 days; twenty five percent responded within one month, 66% within six months and 96% within one year.

### Data collection

Data were collected through a self-administered questionnaire containing 10 questions, of which nine included open-ended alternatives with possibilities to add recommendations for improvement. The questionnaire covered aspects of clinical training, personal health and stress management, leadership styles and knowledge transfer, socio-cultural exposure, and attitudes from others when returning home (see Appendix).

### Measurement instrument

The questionnaire was developed by the IFRC based on their information of interest on how to better prepare Red Cross staff for future viral epidemic outbreaks. This was the IFRC’s first response to this type of mission and it was new to their staff. Before administering, the questionnaire was tested on experienced Senior Health Advisors and Emergency Health Services Senior Officers working within humanitarian organisations for validation of the indicators that were introduced. Furthermore, a pilot questionnaire was tested at the Swedish Red Cross University College (SRCUC) on nurses who had previously been on missions in similar emergency situations. Suggestions from them were to reduce the number of questions and not to pre-define words like ‘adequate’ and ‘coping’, but rather to leave it up to each respondent to interpret their own meaning of the wording.

### Study variables

***Socio-demographic variables*** were age, gender and country of residence.

***Clinical training*** covered aspects of previous humanitarian work experience, previous ERU training (operational training given by the IFRC enabling deployment of emergency medical teams at short notice to a disaster response), and number of times having been on mission at the ETC in Kenema.

***Personal health and stress management*** was measured by questions on health preparation before deployment and health follow up after completed assignment, as well as questions on how the participants coped with and handled stress.

***Leadership styles and knowledge transfer*** were questions focusing on leadership skills, teamwork training and principles of behaviour.

***Socio-cultural exposure*** focused on ability to adapt and work in other cultural and traditional settings than the respondents were normally accustomed to.

***Attitudes from others*** covered reactions they met in their daily environment when returning home after having completed the deployment.

Questions were also asked about the information given to family and friends before deployment. Finally, the participants were asked to write a concluding statement on how they had experienced their deployment.

### Ethical considerations

Research followed the guidelines presented in the World Medical Association Declaration of Helsinki [34] and internationally accepted ethical standards for research, such as autonomy, integrity and confidentiality. An invitation letter was sent to the study population from the IFRC explaining the background, clarifying the purpose and providing a description of the study. If accepting participation, they were asked to send an email to the principle investigator (PI) at the SRCUC stating ‘Yes’. Autonomy and integrity recognises the individuals’ own capacity of decision and the individuals’ right to privacy; the information letter clearly stated that participation was voluntarily and that respondents were entitled to withdraw from the study at any moment without having to give any explanation. The letter also explained confidentiality, meaning that the respondent’s personal identity would be protected at all times and that it would not be possible to trace back the information to a specific individual, neither for the IFRC nor when data are being published. The participant’s identity was replaced by a code number and only the PI at the SRCUC has access to the code key. Researchers performing the analysis only had access to the code number, age and gender. Data were stored in a locked area at the SRCUC, also the academic institution responsible for the data.

Furthermore, the Swedish Act concerning the Ethical Review of Research Involving Humans (2003:460) [35] was followed. The authors also obtained an advisory decision from the Swedish Central Ethical Review Board (CEPN), stating that the present study did not include any sensitive or discriminatory personal data, nor any biological material, nor any apparent risk of injuring the research subject either physically or mentally (see Sections 3 and 4 in the Act). The participants were all experienced nurses and volunteers for a humanitarian organisation; they had given their informed consent and could withdraw from participation at any time without giving any explanation. The study was initiated and approved by the IFRC and the Code of Conduct for the International Red Cross and Red Crescent Movement and Non-Governmental Organisations (NGOs) was discussed and followed in a research context [36].

### Data analysis

Data were analysed using both quantitative and qualitative methods. In the present study, the quantitative data were analysed through chi-square tests for categorical variables and analysis of variance for continuous variables. For these analyses, IBM SPSS Statistics 22 for Windows (IBM, SPSS Inc., Chicago, IL) was used. Data from the open-ended questions were analysed descriptively and complemented by descriptive statistics to describe the study sample [37]. In order to get a comprehensive understanding of the data, the answers of each participant were read several times and encoded by two of the authors. It could consist of one or more statements, representing the content as a whole. Based on similarities and differences, the codes were grouped according to contrast and comparison. A marginal transformation was done during the analysis as this approach allows the researchers to stay close to the data. Such interpretation is straightforward due to its low inference [37]. Specific citations, representative of the findings and illustrating the phenomenon, were then reported in the results.

## Findings

Descriptions of the participants are presented in Table 1. The mean age was 44.9 years, ranging from 25 to 61 years, and the majority of the participants were women (81.8%). The participants came from 15 different countries (i.e. country of residence), representing four different continents: Europe (Belgium, Germany, Norway, Finland, Great Britain, Republic of Ireland, Spain, Sweden and Switzerland), North America (Aruba and Canada), Oceania (Australia and New Zealand), and Africa (Kenya and South Africa). Most of them had previous experience from humanitarian work. However, for 11 participants (25%) this was their first mission. Twenty-six persons (59.1%) reported having had previous ERU training before this mission, whereas 18 (40.9%) had had no such previous training. Ten participants (22.7%) had been in Kenema more than once; of them seven persons had been there twice and three persons had been there three times. Almost 38% of the men and 19% of the women had been more than once in Kenema (p < 0.05), and of them 80% had previous humanitarian work experience.

**Table 1.** Description of the study population; age, gender, country of residence, previous humanitarian work experience and/or ERU training, and number of times in Kenema.

	Frequency
Age	Mean (±SD) 44.9 (10.4)
	Range 25–61
Gender	Women 36 (81.8%)
	Men 8 (18.2%)
Continent of residence	Europe 27 (61.4%)
	North America 5 (11.4%)
	Oceania 10 (22.7%)
	Africa 2 (4.5%)
Previous humanitarian work experience?	Yes 33 (75.0%)
	No 11 (25.0%)
Previous ERU training^a^?	Yes 26 (59.1%)
	No 18 (40.9%)
Number of times in Kenema?	1 34 (77.3%)
	2 7 (15.9%)
	3 3 (6.8%)

Note: ^a^ Emergency Response Unit: operational training enabling deployment at short notice at a disaster response.

In the following section, results are presented both quantitatively as proportions (percentages), and qualitatively through citations representative of the findings.

A majority of the participants considered the pre-deployment health preparation to be adequate (84.1%) (see Table 2). Specifically, they commented on updated vaccination programmes, and the thorough medical check-ups where both present and past health concerns were addressed. The pre-deployment course was described as excellent and included a two-day security and stay safe course. Another positive experience was meeting with nurses who had returned from the area. As two participants commented, ‘it was important for me to talk with the returnees, about their experiences and how they have managed it’, and, ‘it was essential for me to be informed about the risks by the ones who had returned.’ However, one respondent mentioned not remembering having any health preparation before deployment; another wrote that being part of the first deployed team, things were not so clear about how to care for oneself when in the field.

**Table 2.** Percentage distribution among the study population from data covering health issues, stress management, socio-cultural exposure adaptation and attitudes from others after deployment.

	Frequency (%)
**Personal health**	
Pre-deployment health preparation	
Adequate	37 (84.1)
Partially adequate	6 (13.6)
Not good	1 (2.3)
Health follow-up after deployment^a^	
Adequate	36 (83.7)
Not good	7 (16.3)
**Information on health risks and communicable diseases given to family and friends before deployment^a^**	
By the Red Cross	29 (67.4)
Only from participant	12 (27.9)
Received no information	2 (4.7)
**Ability to handle stress**	
Pre-deployment^a^	
No problems	43 (100)
Problematic	0 (0)
During deployment^a^	
Coped well	42 (97.7)
Problematic	1 (2.3)
After deployment^b^	
No problems	36 (85.7)
Problematic	6 (14.3)
**Working in a different socio-cultural environment^a^**	
Adapted well	34 (79.1)
Problematic	5 (11.6)
Not relevant, no contact	4 (9.3)
**Attitudes from others when arriving home after deployment^b^**	
Positive	15 (35.7)
Negative	11 (26.2)
Both	16 (38.1)

Note: ^a^ missing information from one participant; ^b^ missing information from two participants.

A majority also found the personal health follow-up after deployment to be adequate (83.7%). Those who had a negative opinion mentioned not receiving any compulsory medical check-up, and despite being pre-informed that they would be contacted within 24 hours after returning home, it sometimes took one to three weeks. Fifty-two percent recommended a need for improvements, such as improved medical arrangements with local health authorities, and access to an emergency telephone number for returnees from the ETC. Overall they stressed the importance of mental health support combined with psychosocial support after completing the mission. One participant wrote that ‘it is of great importance to have a well-planned mental and health status follow-up upon return … everybody who is deployed should have a health follow-up.’

Sixty-seven percent reported that adequate information was given to relatives, friends, employers and colleagues about the risks associated to the deployment; however, almost 28% stated that this information was supplied only by themselves and not by the employer. There was a discrepancy for those who were deployed during 2014 compared to 2015, as the information given by the IFRC about the Ebola disease and its outbreak improved over time, although the difference was not statistically significant (61.9% *vs* 72.7%).

None reported having problems with handling pre-deployment stress, and only one person reported having had problems handling stress during their time at the ETC (see Table 2), even though it was common to acknowledge the challenging environment. Notably, 14% reported having problems managing stress after returning home. Sixty-one percent suggested need for improvements, and emphasised the importance of monitoring the delegates’ mental health and psychosocial situation before, during and after deployment, as the working environment could be very stressful and potentially traumatic. One delegate wrote: ‘Some images never go away.’ Sometimes stress did not occur until several weeks after having returned home when there was no longer any tailored medical support available. When in Kenema, they said, ‘the adrenalin was kicking’, and they were confident about their professional performance, their capacity to adapt and their flexibility. Some suggested regular phone contact with psychologists after deployment. One participant even suggested, ‘it would have been good to have a 24-hour health support hotline upon return.’ Another commented, ‘follow up calls as per 3 days upon return would be helpful … some stress was not realized until I’d been home a few weeks and there was no longer support in place’.

Some reported difficulties working together with local health care staff due to different understanding and perception regarding the concepts of severity, priority and urgency. Also, the ‘no touch’ approach had a more flexible and less strict meaning for the local as compared to the expatriate staff. Almost 80% reported having adapted well to working in a different socio-cultural environment than they were accustomed to. Many referred to previous experiences working in various socio-cultural environments, having learnt to respect and adapt to the current circumstances. One quote from a participant summarises well the state of mind of most international staff:

I always listen first and watch how the national staff behaves and acts. As a second step, I try to improve the service and adapt my communication according to what I saw. I always try to see things positive and acknowledge what great job the national staff does.

The participants reported various reactions from others when returning home after deployment; 35.7% received only negative comments and 26.2% only positive comments, whereas 38.1% experienced mixed and polar reactions. One delegate wrote that the reactions shifted ‘from being a hero to pariah’. One general comment was that people were making jokes of whether they could be touched or not, and this was not at all appreciated by the nurses. One participant wrote: ‘I received mostly praise from people, but also a lot of bad jokes were made such as me being contagious but it was mostly meant in a humorous way.’ Others reported that they were met with fear; people were afraid that they could be transmitting the disease, and therefore they had to hide having been at an ETC mission. For instance, one participant shared: ‘I experienced negative comments and behaviours from friends. My re-entry into the country was low profile compared to other colleagues … not wanting to share gym equipment, swim in the same pool … ’ However, people also expressed gratitude for fighting the disease.

Table 3 presents data on leadership management and knowledge transfer. Thirty-eight percent reported having received adequate leadership training for working in an acute epidemic outbreak, whereas 33.3% had not. The remaining 28.7% reported it as irrelevant as they did not have a senior position at the ETC. A majority (92.9%) described themselves as good leaders, and reported having previous leadership experience from their profession as nurses. When asked to describe which leadership skills they considered most needed during an acute epidemic outbreak, three leadership styles were shown: democratic (75.7%); authoritarian (16.2%); and task-oriented (8.1%).

**Table 3.** Percentage distribution among the study population from data on leadership management and knowledge transfer.

	Frequency n (%)
Did you receive adequate leadership training before deployment?^a^	
Yes	16 (38.1)
No	14 (33.3)
Not relevant, did not work as a leader	12 (28.6)
Describe your leadership skills^a^	
Good	39 (92.9)
Poor	2 (4.8)
Not relevant	1 (2.4)
Describe appropriate leadership skills for Ebola outbreaks^b^	
Democratic	28 (75.7)
Authoritarian	6 (16.2)
Task-oriented	3 (8.1)
Importance of pre-deployment knowledge transfer^a^	
Relevant	34 (81.0)
Insufficient	8 (19.0)
Importance of pre-deployment training on teamwork, principles and behaviour^c^	
Useful	38 (88.4)
Insufficient	5 (11.6)

Note: ^a^ missing information from two participants; ^b^ missing information from three participants; ^c^ missing information from one participant.

The pre-deployment knowledge transfer was reported to be relevant by 81%, and the pre-deployment training on teamwork, principals and behaviour as useful by 88.4% (see Table 3). Fifty-four percent suggested improvements, and many respondents referred to the Disaster Management Information System (DMIS) as an excellent tool for knowledge transfer. DMIS is a web-based working tool made accessible to Red Cross and Red Crescent staff working in National Societies, delegations and Geneva headquarters. Others referred to the need for more clinical knowledge, such as waste management and cleaning and disinfection procedures, as well as practical training on how to dress/undress safely, as the correct order for donning (putting on) and doffing (taking off) the Personal Protective Equipment (PPE) is vital in controlling the spread of infectious diseases such as Ebola. They also commented on the lack of practical sessions on dead body management. Some of them also acknowledged the lack of time to prepare themselves, both mentally and clinically: ‘not enough time to prepare for everything we should know’.

The nurses who arrived at the ETC in 2014 had significantly more experience in previous humanitarian work and ERU-training compared to those arriving in 2015 (86.4% *vs* 63.6% and 72.7% *vs* 45.5% respectively; p < 0.05), see Figure 1. Nonetheless, they reported a higher prevalence of problems with handling stress after deployment (22.7% *vs* 5.0% respectively; p < 0.05).

In Figure 2, the recommendations as suggested by the participants are summarised on how to better prepare health care staff for future VHF outbreaks. **Before deployment**, they reported a need for more practical exercises specific for the task, and relevant information on facts adapted to this specific kind of deployment, to-do lists and so on. The importance of learning from previous returnees’ experiences was also mentioned. They wanted more in-depth information to give to family and colleagues back home. **During deployment**, they reported a need for interpersonal contacts, teamwork and strong group dynamics. Most important was professional trust between colleagues and positive work dynamic. Reduction of the workload and more time off was expressed as necessary, as exhaustion increases the risk of mistakes. Participants shared a common trust and pride in working for the Red Cross movement. **After deployment**, they stressed the need for mental health and psychosocial support, and they requested deeper knowledge about coping strategies. The respondents reported being focused on their duties and safety during deployment, and only allowing emotional reactions afterwards. They also stressed the importance of active social contact and support between colleagues.

## Interpretations

This study aimed to explore how nursing staff experienced their deployment before, during and after having worked at an ETC, and to supply knowledge on how to better prepare health care staff for future VHF outbreaks. The main findings can be summarised as follows:

In general, the participants:

- Were satisfied with their pre-deployment physical health preparation, whereas they stressed the importance of mental health support combined with psychosocial care after deployment;
- Expressed a need for more pre-deployment clinical task specific exercises, as well as targeted information adjusted to the explicit mission;
- Described themselves as good leaders, and preferred a democratic style of leadership that required guidance but was still controlled by a specific leader;
- Defined a need for interpersonal contacts, teamwork and strong group dynamics;
- Reported mixed reactions from others when returning home: ‘from being a hero to pariah’.

### Pre-deployment

The nurses were satisfied with their pre-deployment training, and the comments ranged from good to excellent, as has also been reported by others [25]. However, they also commented that they would have liked more practical sessions, especially on dead body management, waste management, cleaning and disinfection procedures, as well as nursing care under these specific conditions [15]. As reported by others [38,39], nurses do not feel adequately prepared as there is not sufficient formal training in disaster management, and identified a range of practical learning needs. Although, in this study, practical preparatory training was offered via ERU, not all nurses participated in such a course. The participants also emphasised the need for additional information on infectious diseases and on living conditions, specifically first-hand information from the returnees about what was characteristic for that particular mission. They wanted more documented in-depth information to provide to family and colleagues back home, as they themselves had had to be the direct providers of such information. However, they were aware that it was the first time that the IFRC was sending medical staff dealing with a VHF outbreak, and they trusted the organisation despite the situation.

Our results confirm the need to integrate disaster-management, or emergency-risk management, training into nursing education programmes as reported by others [14–18]. Increased globalisation, and nurses being the major contributors to global health care, states the need for health services to be prepared to cope with major emergencies or disasters, as well as acts of terrorism [40]. The past decades have brought some of the worst VHF outbreaks and natural disasters ever, and a simple act of terrorism can cause unprecedented destruction and death. In fact, the epidemiological transition is clearly showing an increasing trend in infectious disease and epidemics worldwide due to multiple factors such as wars, natural disasters, resistance to antibiotics, travelling, poverty, rapid urbanisation, forced migration and so on [41,42].

### During deployment

For some respondents, leadership training was not seen as relevant as they had not had the position of a leader during their missions. They emphasised team work and team dynamics as being more important than leadership skills. According to them, a good leader should be democratic and a good listener, but at the same time strict about safety. Previous research has found that a democratic leadership style is one of the most effective and creates higher productivity, better contributions from group members and increased group morale [43]. A democratic leadership refers to sharing ideas and policies; members of the group take a more participative role in the decision process. Nevertheless, while democratic leadership is one of the most effective leadership styles, it does have some potential downsides, and in situations where roles are unclear or time is of the essence, democratic leadership can lead to communication failures [43]. This was confirmed in the present study, as it was reported by some that lack of firm leadership led to failed empowerment of staff members and problems keeping up the morale of the team. In addition, a quick turnover of leadership made things difficult, especially for the locals.

The nurses stressed the importance of interpersonal communication on site as they were coping by talking to others, laughing and crying together. They expressed that they knew what to do and they performed, describing themselves as professionals, but saw reducing the workload as a priority, as when the nurses got too tired they tended to make more mistakes. This is in accordance with studies by Abbasi et al. [44] and Andertun et al. [25], who found that the work ability of nurses improved by including pre-employment examinations, proper management of work-shift conditions, and using engineering and administrative strategies to ensure the safety of hospitalised patients.

Participants reported that inter-cultural adaptation was not an issue, as they were used to working in different socio-cultural environments. Although cultural and social sensitivity was not an objective of this study, it would be interesting to explore how the locals perceived that the international nurses adapted to working in a different culture, and their own experiences of working with nurses who were expatriates.

### After deployment

The participants strongly emphasised the need for additional mental health and psychosocial support and more knowledge about coping strategies. Some described being focused during deployment and only allowing emotional reactions afterwards as an after shock period or a hangover. It has been reported by others that team members rely on each other for support in coping with the traumatic experiences at a disaster site [11], and for 11 nurses this was their first mission. It is notable however that the most experienced nurses, who had been on several previous missions, were the ones who reported having the highest mental stress after deployment. This could be because they were working at the ETC during the first period and therefore had to build up the centers, meeting the first cases and trauma, and so on. This is important information for employers, as they might assume that the nurses’ previous experiences will protect them and therefore give them less attention [23].

Another aspect is the attitudes of others when returning home after completed missions [26]. Many blamed the media that according to them reported sensational news and not many facts, as well as poorly managed information by the health communities that led to unnecessary fear and anxiety among the public.

### Nurses volunteering to work in dangerous situations

The EVD outbreak is a reminder that nursing may be a risky profession. This is, however, nothing new. Nurses have through history placed themselves in potentially hazardous situations when providing care for others: from the plague in the Middle Ages to typhoid fever in Florence Nightingale’s time to AIDS in recent decades [45]. Many health care workers, including nurses, also became victims of the disease (EVD) they were trying to defeat [28,29,46]. Why does one volunteer to work in dangerous situations, as many in our study have chosen repeatedly to do? Zinsli & Smythe [23] suggest that it is the *sameness* in the human-to-human call and response to need that holds nurses in such work, whereas it is the *difference* s/he encounters, i.e. personal danger, extent of injuries, limits of treatment, and overwhelmingness of need that will shape a life-changing, and in the worst case, life-claiming, experience [23]. Further research is needed on how to best prepare, educate and understand the experience, often traumatising, of nurses volunteering to work in dangerous situations, as well as how to meet them upon their return after deployment.

One may expect, already in the near future, a global increase in frequency and intensity of outbreaks similar to EVD, recurrent disasters and emergencies requiring global nursing. In other words, it is in the interest of the world community to equip most efficiently those highly specialised humanitarian care workers.

### Methodological considerations and limitations

This is, to our knowledge, one of the few investigations studying nurses’ own self-reported experiences related to their involvement at the ETC during a VHF outbreak. A major strength of the study is the combined qualitative and quantitative approach with a possibility for longitudinal follow-up. Another strength is the multidisciplinary profile of the authors, allowing a comprehensive approach to this study.

Almost 17% of the email addresses received from the IFRC were not valid, and this shows the difficulties in contacting nurses who accept temporary humanitarian assignments. We had email contact with five of the six nurses who did not respond to the questionnaire (drop-outs). They were out on new missions and reported having poor internet connections. In the end, a deadline had to be set.

No pre-existing validated protocols were used in this study, as the questionnaire was developed by the IFRC with the objective to find out how to best prepare their staff for future viral epidemic outbreaks. This could be seen as a limitation; however, we argue that the questions reflect the objectives of the study. The responses were given on the basis of self-assessment, and it was left to the respondents to interpret the meaning or the wording, i.e. if something was *adequate* to them or not. We believe that the results can be transferred to similar contexts, and there was great consistency in the answers of the respondents. Discrepancies in the results were due to early (in 2014) or late (in 2015) deployment, as it was more common for nurses active in 2014 to have previous humanitarian experience and ERU training compared to nurses active in 2015.

## Conclusions

Participants were generally satisfied with their deployment, but stressed the importance of mental health support combined with psychosocial support after deployment; the need for more specific ERU training was also recurrent. An active dialogue and communication with colleagues were perceived as important, and information given to family and colleagues was found to be relevant but not sufficient. Furthermore, a reduction of the workload was requested to reduce the risk of making mistakes. One may expect, already in the near future, an increase globally in frequency and intensity in outbreaks similar to EVD, recurrent disasters and emergencies requiring global nursing. In other words, it is in the interest of the world community to equip most efficiently and effectively those highly specialised humanitarian care workers.

### Recommendations for the future

Mental health support combined with psychosocial support, and hands-on coping strategies with focus on pre- and post-deployment;

1. Specific task-oriented clinical training;
2. Workload reduction, as exhaustion is a risk for safety;
3. Relevant, adequate and updated information about EVD given to family and colleagues back home.

## Paper context

Nurses are on the forefront and play a key role of global disaster responses. Nevertheless, they are often not prepared for the challenges they are facing and research is scarce regarding the nursing and coping skills required for first responders needed during a disaster situation. This study supplies knowledge and gives recommendations on how to better prepare health care staff for future viral haemorrhagic fever outbreaks and other disasters.

## Appendix

Global nursing in acute viral epidemic outbreaks: before, during and after deployment

A joint research project between the Swedish Red Cross University College (SRCUC) and the International Federation of Red Cross and Red Crescent Societies (IFRC).

Please, write your answers below each question, or if you use a separate paper, make sure to include the corresponding question number next to your answer for identification purposes.

**1. Background information**

a) How old are you?

b) What is your gender?

c) In which country do you reside?

d) How were you recruited for this deployment: via the RC ERU or from another source?

e) Did you have any previous RC ERU training(s) before this deployment?

f) Do you have any previous humanitarian work experience?

g) How many times have you been deployed at the Ebola Treatment Center (ETC) in Kenema?

h) When did your deployment at the ETC end? (Write which month and year. If more than once, please provide the last.)

**2. Health concerns**

a) Did you find that your personal health preparation by the RC was adequate?

–If yes, why was it adequate?

–If no, why not?

b) Did you find that your personal health follow-up by the RC was adequate?

–If yes, why was it adequate?

–If no, why not?

c)Did your relatives/friends/employers/colleagues receive adequate information and an opportunity to voice their concerns before, during and after your deployment?

d) What improvements would you suggest to the RC in regards to personal health management?

**3. Handling stress**

a) How do you cope and handle stress? (i.e. dealing with traumatic events, risk of contamination, operating in draining environments and people’s attitudes towards you when returning home)

– Pre-, during and after deployment. Please give examples.

b) How could the RC improve its support for stress management to medical staff working in this type of operation?

**4. Socio-cultural environment**

How did you adapt your work in an environment with different understanding of health and cause of illness, and relating to traditional healers and priests?

**5. Teamwork, principles and behavior**

a) How do you consider the relevance of the pre-deployment training on teamwork, principles and behavior?

b) What are the changes you would suggest the pre-deployment training to make in order for your work to become more effective and relevant?

**6. Leadership and management**

a) Did the RC provide adequate leadership training for working in an acute epidemic outbreak?

–If yes, what was adequate?

–If no, what was not?

b) How would you describe your leadership skills in acute epidemic outbreaks?

c) What management styles do you consider most appropriate for this type of operation?

**7. Knowledge transfer**

a) How do you consider the pre-deployment knowledge transfer (sufficiently deep, wide, relevant)?

b) If you were to plan and organize the pre-deployment knowledge, which changes would you implement?

**8. Attitudes**

What kind of reactions have you met in your daily environment when returning home after having worked in an acute epidemic outbreak?

**9. Conclusions**

How would you consider, in general, your preparation and training by the RC compared to health workers working for other humanitarian organizations?

**10. Miscellaneous**

Please feel free to add any comments you would like.

Thank you for your participation!
